# Integrated Bioinformatics Analysis of mRNAs and miRNAs Identified Potential Biomarkers of Oral Squamous Cell Carcinoma

**DOI:** 10.31557/APJCP.2020.21.6.1841

**Published:** 2020-06

**Authors:** Nasrin Amiri-Dashatan, Mehdi Koushki, Ali Jalilian, Nayeb Ali Ahmadi, Mostafa Rezaei-Tavirani

**Affiliations:** 1 *Proteomics Research Center, Shahid Beheshti University of Medical Sciences, Tehran, Iran. *; 2 *Department of Clinical Biochemistry, Faculty of Medicine, Tehran University of Medical Sciences, Tehran, Iran. *; 3 *Proteomics Research Center, Faculty of Paramedical Sciences, Shahid Beheshti University of Medical Sciences, Tehran. *

**Keywords:** Oral squamous cell carcinoma, microarray analysis, DEGs, miRNA, protein, protein interaction network

## Abstract

**Background:**

Oral cancer is a frequently encountered neoplasm of the head and neck region, being the eighth most common type of human malignancy worldwide. Despite improvement in its control, morbidity and mortality, rates have improved little in the past decades. The present investigations about gene interaction and pathways still could not clear the appearance and development of oral squamous cell carcinoma (OSCC), completely. The aim of this study is to investigate the key genes and microRNAs interaction in OSCC.

**Materials and Methods:**

The microarray datasets GSE13601 and GSE98463, including mRNA and miRNA profiles, were extracted from the GEO database and were analyzed using GEO2R. Functional and pathway enrichment analyses were performed by using the DAVID database. The protein–protein interaction (PPI) network was constructed and analyzed using STRING database and Cytoscape software, respectively. Finally, miRDB was applied to predict the targets of the differentially expressed miRNAs (DEMs).

**Results:**

Totally, 97 differentially expressed genes (DEGs) were found in OSCC, including 66 up-regulated and 31 down-regulated genes. The gene ontology (GO) and Kyoto Encyclopedia of Genes and Genomes (KEGG) pathway enrichment analyses showed that up-regulated genes were significantly enriched in movement of cell or subcellular component, cell adhesion, biological adhesion, cellular localization, apoptotic signaling pathway, while the down-regulated genes were enriched in muscle system process and oxidation-reduction process. From the PPI network, the top 10 nodes with the highest degree were detected as hub genes. In addition, 18 DEMs were screened, which included 7 up-regulated and 11 down-regulated miRNAs. STAT1 was potentially targeted by three miRNAs, including has-miR-6825-5P, has-miR-4495, and has-miR-5580-3P.

**Conclusion:**

The roles of DEMs such as hsa-mir-5580-3p in OSCC through interactions with DEGs CD44, ACLY, ACTR3, STAT1, LAMC2 and YWHAZ may offer a suitable candidate biomarker pattern for diagnosis, prognosis and treatment processes in OSCC.

## Introduction

Oral squamous cell carcinoma (OSCC) is the sixth most common cancer type worldwide. Despite advances in treatment and surgical methods, the 5-year survival rate for patients with oral cancer did not show improvement in the last two decades (Malik et al., 2016). The incidence of OSCC is also increasing in recent years, especially in younger people. Therefore, further studies are needed to better understanding of molecular mechanisms underlying this disease. Recently, several studies have reported that changes of gene expression levels is related to formation and progression of OSCC tumors, however, there is an urgent need to identify potential mRNA biomarkers for prediction, early diagnosis and treatment of oral tumors (Chakraborty et al., 2010; Ge et al., 2015). MicroRNAs (miRNAs) are small, noncoding RNAs with 18–25 nucleotides long that regulate protein expression at the post-transcriptional level. MiRNAs involvement in various physiological processes including cell proliferation, differentiation, apoptosis, and angiogenesis has been reported (Malik et al., 2016; Park et al., 2017). The abnormal expression of specific miRNAs can lead to cancer progression (Momen-Heravi and Bala, 2018). The use of microRNAs as diagnostic and prognostic markers in diseases has been considered in recent years. Several studies have reported miRNAs profiling and differentially expressed miRNAs in OSCC compared to normal tissue (Chen et al., 2008; Manikandan et al., 2016; Schneider et al., 2018). Today, bioinformatics and computational methods play an important role in biological studies, especially in protein and gene regulatory network analysis that led to identify key genes and proteins in different diseases pathogenesis (Atan et al., 2014; Atan et al., 2018; Dashatan et al., 2018). This study aimed to assess the mRNA-miRNA network and pathway enrichment analysis on extracted genes and miRNAs from GEO database to find potential molecular mechanisms associated with oral cancer.

## Materials and Methods

*Microarray data collection*

 We searched the GEO database (https://www.ncbi.nlm. nih.gov/geo/) using the following keywords: “Oral Squamous cell carcinoma” (study keyword), “Homo sapiens” (organism), “Expression profiling by array” (study type), and “tissue” (attribute name). After a systematic review, two gene expression profiles (*GSE13601* and *GSE98463*) were collected for analysis. Above all, the differentially expressed mRNAs between patients with oral squamous cell carcinoma (OSCC) tissue (n=31) and in those with normal tongue tissue (n=26) were identified by GEO2R from the GSE13601 microarray. The differentially expressed miRNAs between oral squamous cell carcinoma (OSCC) (n=8) and oral mucosa (n=8) were identified by GEO2R from the GSE98463 microarray dataset (Table 1).

*Differential expression of miRNAs and mRNAs*

After GEO2R was applied to analysis differentially expressed mRNAs and miRNAs between OSCC and normal tissue, the differentially expressed mRNAs were selected with a criterion of adj. p<0.05 and log fold change (FC) ≥2.

*Functional annotation and pathway enrichment analysis*

Gene ontology (Go) including biological pathway, molecular function, and cellular component and KEGG pathway analyses of the DEGs were performed using the DAVID database. 

*Protein- protein interaction network analysis*

DEGs (FC>2 and P<0.05) were used for protein interaction network instruction. Protein- protein interaction network was constructed by using STRING database (http://string-db.org). The protein-protein interaction network was visualized and analyzed using the Cytoscape software version 3.6.0. Cytoscape is an open source software that integrates biomolecular interaction networks with large-scale expression data into a unified conceptual framework. The top 10 high node degree genes was screened as hub genes. Clustering analysis of protein network was performed in order to detect modules by using MCODE (Molecular Complex Detection) algorithm with haircut on, node score cut-off = 0.2, k-core = 2, and max. Depth = 100. 

*Prediction of miRNAs targets*

The target genes of the DEMs from GSE98463 were predicted with TargetScan Human database (www.targetscan.org), which is an online database for predicting microRNA targets.

## Results

*Identification of DEGs*

A total of 97 (including 66 up-regulated and 31 down-regulated) genes and 18 (including 7 up-regulated and 11 down-regulated) miRNAs with a threshold p-value<0.05 and FC≥2 were considered as significantly differentially expressed (Supplementary Table 1). The full list of the genes contained in these datasets is shown in Table 1. The most significantly down-regulated mRNA was MYOC (Supplementary Table 1). Significant up-regulation of MMP1 was detected in OSCC tissue samples compared with normal tongue tissue. According to supplementary Table 2, has-miR-1290 and has-miR-5580-3p were detected as significantly up-regulated and significantly down-regulated miRNAs between OSCC samples compared with normal healthy sample.

*Functional and pathway enrichment analyses*

According to Table 2, the GO term enrichment analysis showed that in biological processes-associated category, the up-regulated genes were significantly enriched in movement of cell or subcellular component, cell adhesion, biological adhesion, etc, while the down-regulated genes were enriched in muscle system process and oxidation-reduction process. In addition, the cellular component analysis showed that adherence junction and mitochondrial membrane were mainly enriched category in up-regulated and down-regulated genes, respectively. Moreover, for molecular function, the up-regulated genes were enriched in actin binding, and the down-regulated genes were enriched in cofactor binding and DNA binding. As shown in Table 2, the KEGG pathway analysis indicated that the micoRNAs in cancer, pathway in cancer, FC gamma R-mediate phagocytosis were mainly enriched pathways in up-regulated genes, while two pathways including phenylalanine metabolism and tyrosine metabolism were overrepresented in down-regulated genes.

*PPI network and modules analysis*

**Figure 1:**
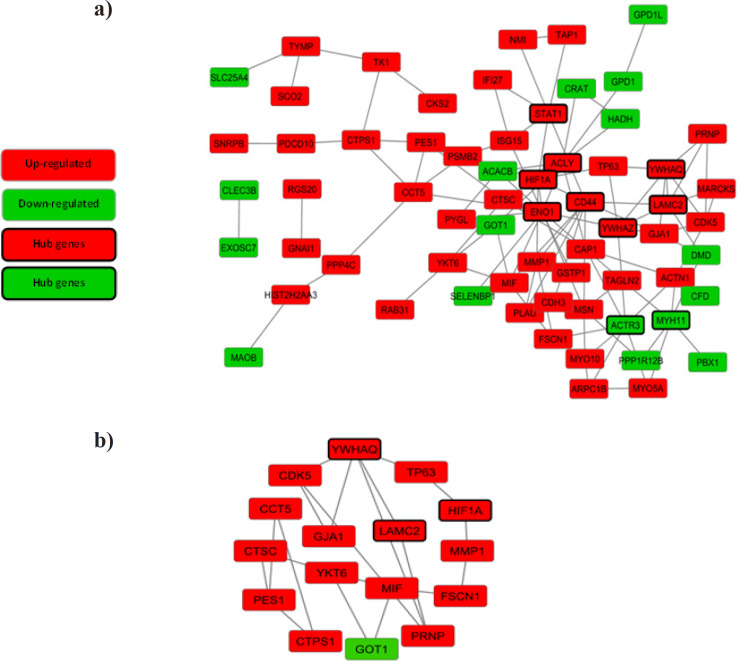
Protein–Protein Interaction (PPI) Network and Hub Genes. a) PPI network of differentially expressed genes (DEGs). b) A significant module selected from the PPI network. Red nodes denote up-regulated genes, while green nodes denote down-regulated genes. Black border shows that the gene is a hub. The lines represent an interaction relationship between the nodes

Sixty-five nodes and 108 edges were mapped in the PPI network of identified DEGs, including 48 up-regulated genes and 17 down-regulated genes (Figure 1a). The top 10 nodes with the higher degrees screened as hub genes are *ENO1, CD44, ACLY, ACTR3, STAT1, MYH11, LAMC2, YWHAZ, YWHAQ*, and *HIF1A* as shown in Table 3. A significant module including 16 nodes and 20 edges was obtained using MCODE which shown in Figure 1b. Biological process enrichment analysis results of module genes have shown in Table 4.

*miRNAs targets results*

In this study, top 10 DEMs were identified including 5 up-regulated miRNAs and 5 down-regulated miRNAs. The TargetScan Human database was used to predict target genes of the identified DEMs as shown in Table 5. The has-miR-1290, one of the significantly up-regulated miRNAs, was found to target *ACTR3*, *MYH11*, and *YWHAZ*. At the other hand, has-miR-5580-3p, the main down-regulated miRNAs, potentially targeted *CD44, ACLY, ACTR3, STAT1, LAMC2 *and *YWHAZ*. Moreover, we found that *ACLY, STAT1* and *YWHAZ* was potentially targeted by three miRNAs (Table 5). 

**Table 1:** Characteristics of mRNA and miRNA Expression Profiling of Oral Squamous Cell Carcinoma (OSCC)

GEO ID	Platform	Samples (cases/controls)	Country	References
GSE13601	GPL8300 [HG_U95Av2] Affymetrix Human Genome U95 Version 2 Array	31/26	USA	Estilo CL et al. (Estilo et al., 2009)
GSE98463	GPL21572 [miRNA-4] Affymetrix Multispecies miRNA-4 Array [ProbeSet ID version]	8/8	Spain	Chamorro-Petronacci C et al. (Chamorro-Petronacci et al., 2018a)

**Table 2:** Functional and Pathway Enrichment Analysis of Up-Regulated and Down-Regulated Genes in Oral Squamous Cell Carcinoma (OSCC) Tissue

Category	Term	Count	*P*-value
Up-regulated			
GOTERM_BP_FAT	GO:0006928~movement of cell or subcellular component	20	5.70E-06
GOTERM_BP_FAT	GO:0007155~cell adhesion	19	1.10E-05
GOTERM_BP_FAT	GO:0022610~biological adhesion	19	1.10E-05
GOTERM_BP_FAT	GO:0051641~cellular localization	23	2.10E-05
GOTERM_BP_FAT	GO:0097190~apoptotic signaling pathway	11	2.90E-05
GOTERM_CC_FAT	GO:0005912~adherens junction	19	1.00E-10
GOTERM_CC_FAT	GO:0070161~anchoring junction	19	1.50E-10
GOTERM_CC_FAT	GO:0070062~extracellular exosome	30	1.50E-07
GOTERM_CC_FAT	GO:1903561~extracellular vesicle	30	1.60E-07
GOTERM_CC_FAT	GO:0043230~extracellular organelle	30	1.70E-07
GOTERM_MF_FAT	GO:0003779~actin binding	11	1.90E-06
GOTERM_MF_FAT	GO:0008092~cytoskeletal protein binding 15	15	2.00E-06
GOTERM_MF_FAT	GO:0051015~actin filament binding	7	9.70E-06
GOTERM_MF_FAT	GO:0050839~cell adhesion molecule binding	10	5.00E-05
GOTERM_MF_FAT	GO:0098641~cadherin binding involved in cell- cell adhesion	7	7.30E-04
KEGG_PATHWAY	hsa05206:MicroRNAs in cancer	6	2.40E-02
KEGG_PATHWAY	hsa05200:Pathways in cancer	7	2.50E-02
KEGG_PATHWAY	hsa04666:FC gamma R-mediated phagocytosis	3	8.50E-02
KEGG_PATHWAY	hsa04512:ECM-receptor interaction	3	9.10E-02
Down-regulated			
GOTERM_BP_FAT	GO:0003012~muscle system process	6	3.60E-04
GOTERM_BP_FAT	GO:0055114~oxidation-reduction process	8	6.00E-04
GOTERM_BP_FAT	GO:0010765~positive regulation of sodium ion transport	3	1.40E-03
GOTERM_BP_FAT	GO:0006732~coenzyme metabolic process	5	1.40E-03
GOTERM_BP_FAT	GO:0006082~organic acid metabolic process	3	2.00E-03
GOTERM_CC_FAT	GO:0031966~mitochondrial membrane	8	1.60E-04
GOTERM_CC_FAT	GO:0005743~mitochondrial inner membrane 7	7	2.00E-04
GOTERM_CC_FAT	GO:0005740~mitochondrial envelope	8	2.30E-04
GOTERM_CC_FAT	GO:0019866~organelle inner membrane	7	3.70E-04
GOTERM_CC_FAT	GO:0005739~mitochondrion	11	4.20E-04
GOTERM_MF_FAT	GO:0048037~cofactor binding	6	8.00E-05
GOTERM_MF_FAT	GO:0051287~DNA binding	4	9.30E-05
GOTERM_MF_FAT	GO:0050662~coenzyme binding	5	2.40E-04
GOTERM_MF_FAT	GO:0004367~glycerol-3-phosphate dehydrogenase activity	2	3.40E-03
GOTERM_MF_FAT	GO:0016616~oxidoreductase activity	3	1.60E-02
KEGG_PATHWAY	hsa00360:Phenylalanine metabolism	2	4.60E-02
KEGG_PATHWAY	hsa00350:Tyrosine metabolism	2	9.20E-02

**Table 3:** Top 10 Hub Genes and Related DEMs

Gene symbol	Node degree	Related DEMs
*ENO1*	12	has-miR-6825-5P
*CD44*	9	has-miR-7111-5P, has-miR-5580-3P
*ACLY*	8	has-miR-7111-5P, has-miR-509-3-5P, has-miR-5580-3P
*ACTR3*	7	has-miR-1290, has-miR-5580-3P
*STAT1*	6	has-miR-6825-5P,has-miR-4495, has-miR-5580-3P
*MYH11*	6	has-miR-4495, has-miR-1290
*LAMC2*	6	miR-5580-3P
*YWHAZ*	6	has-miR-6825-5P, has-miR-1290, miR-5580-3P
*YWHAQ*	6	has-miR-6825-5P, has-miR-509-3-5P
*HIF1A*	6	has-miR-4495, has-miR-509-3-5P

**Table 4:** Functional (Biological Process) Enrichment Analysis of the Genes in the Module

Category	Term	Count	*P*-value
GOTERM_BP_FAT	GO:0044085~cellular component biogenesis	7	9.70E-03
GOTERM_BP_FAT	GO:0002070~epithelial cell maturation	2	1.50E-02
GOTERM_BP_FAT	GO:0051130~positive regulation of cellular component organization		1.50E-02
GOTERM_BP_FAT	GO:0045926~negative regulation of growth	5	1.50E-02
GOTERM_BP_FAT	GO:0010941~regulation of cell death	3	2.60E-02
GOTERM_BP_FAT	GO:0006915~apoptotic process	5	3.20E-02

**Table 5:** Significantly DEMs in OSCC Tissue and Their Potential Target Genes

miRNAs	logFC	Target Genes
has-miR-let-7d-3p	0.79	*PARP11, HMGA2, SH3RF1, MEX3C, SEC24D, BEND2, PTGIS, PRKACB, GSTK1, PHF14*
has-miR-6825-5p	0.93	*NACC1, ENO1, BACH2, RIMS3, VAT1, STAT1, BCAS1, CDK18, YWHAZ, YEHAQ*
has-miR-7111-5p	1.01	*PPP1R9B, CD44, WIZ, SLC6A17, NFIX, NR1D1, ACLY, ELK1, SRF, CASTOR2*
has-miR-4495	1.15	*MYH11, PIAS2, HIF1A, TMEM33, EXTL2, STAT1, PIEZO2, AZIN1, GPR173, IKZF2*
has-miR-1290	1.36	*SGO1, ACTR3, RTKN2, OSBPL6, ACER3, MYH11, AKAP2, YWHAZ-AKAP2, GTF2I*
has-miR-509-3-5P	-1.26	*CALD1, SERTAD2, AGFG1, ACLY, HIF1A, YWHAQ, DOCK3, CCSER2, CD200R1, FIGN*
has-miR-617	-1.62	*RFXAP, CEP350, STXBP1, DGKH, CMC1, PDGFRL, EVI2A, PRPS2, AKT3, PPA1*
has-miR-509-3P	-1.43	*ST3GAL2, OSBP, DDAH1, MMD2, PIP5K1B, VEZF1, RAB5C, RNF130, HABP4, RFX3*
has-miR-6510-3p	-1.9	*HDAC3, SMURF2, NDFIP1, TSPAN17, AEN, ARID1B, C17orf107, CTIF, PCSK6, MRS2*
has-miR-5580-3p	-1.92	*CD44, BTAF1, ACLY, DENR, ACTR3, STAT1, RASSF8, LAMC2, PDE10A, YWHAZ*

FC, fold change; Positive logFC values denote up-regulated miRNAs, while negative logFC values denote down-regulated miRNAs. If there were more than ten genes predicted by miRDB, only ten genes were listed in the Table

## Discussion

Despite the increasing investigations on OSCC, the early diagnosis and treatment of it is still an issue due to the lack of understanding of the molecular and cellular mechanisms that drive the occurrence and progression of OSCC. Therefore, the need for in-depth studies into the molecular mechanisms of OSCC development is increasingly felt for OSCC prediction, early diagnosis and treatment. In current study, a total of 97 DEGs were identified. By constructing the PPI, we identified high degree genes including S*TAT1, ACLY, HIF1A, ENO1, CD44, YWHAQ, YWHAZ, LAMC2, ACTR3* and MYH11, which among them *ACTR3* and *MYH11* were down-regulated genes and the others were up-regulated ones.

*STAT1 *as another hub gene is the main component in interferon (IFN)-signaling that is involved in cellular functions in response to cytokines. Some clinical studies reported that* STAT1* apply tumor promoter effects (Zhang and Liu, 2017). Moreover, there are also some reports claiming that STAT1 activation in squamous cell cancer of the oral cavity is a potential predictive marker of response to adjuvant chemotherapy (Laimer et al., 2007). In this study, ATP citrate lyase (ACLY) has also been introduced as another hub gene that is involved in lipid biogenesis linked with glucose metabolism. Studies have reported a tumorigenesis role for ACLY. In several cancer types (Migita et al., 2008; Qian et al., 2015; Teng et al., 2018), the ACLY has been expressed in higher levels in comparison to normal cells and its inhibition is known to induce proliferation arrest in cancer cells both in vitro and in vivo (Zaidi et al., 2012). There are also evidences that present ACLY as a promising target for cancer treatment. Zhi et al., (2012) found that patients with tumors located in the oral cavity showed higher levels of *ACLY* gene in the tumor tissues compared to the healthy tissues (Zhi et al., 2015).

Another gene introduced as hub gene in this study was hypoxia-inducible factor-1 (HIF-1) that has been identified as a significant cancer drug target. Recent studies have found evidences of strong correlation between increased levels of HIF-1 and tumor metastasis, angiogenesis, and tumor resistance therapy (Masoud and Li, 2015). Recent researches in cancer biology at the cellular and molecular levels claimed that the HIF-1α pathway is a main survival pathway for which novel strategies of cancer therapy could be developed. There is also a recent meta-analysis study demonstrated that HIF-1α overexpression is associated with tumor size, tumor stage, lymph node metastasis, and overall survival of patients with OSCC. However, HIF-1α could be used as an independent prognostic marker in patients with OSCC (Eckert et al., 2010; Zhou et al., 2017). 

Another hub gene named *ENO1* is also play an important role in tumorigenesis, cancer invasion, and metastasis (Capello et al., 2011; Gao et al., 2013; Zhao et al., 2015; Dai et al., 2017). regarding oral cancer, Ito et al., investigated differential expression of the human *ENO1* gene in oral epithelium and OSCC, and results indicated that differential subcellular localization of *ENO1 *products may be closely related to carcinogenesis of the oral epithelium (Ito et al., 2007). 

Another hug gene, C*D44*, is a non-kinase transmembrane glycoprotein that has various roles in cellular process such as cell division, migration, adhesion, and signaling. *CD44* has been shown to be involved in tumor growth and metastasis and has also been reported as a cancer stem cell (CSC) marker in head and neck squamous cell cancer (HNSCC) (Chen et al., 2014). A study assessed immunohistochemical expression of *CD44* in different grades of OSCC to evaluate its role in cancer progression showed an altered expression level of *CD44* in OSCC with weak immunostaining in poorly differentiated squamous cell carcinoma. Also, they suggested that the loss of cell adhesion which is correlated to the decrease of CD44 expression might be used in determination of OSCC progression (Kaza et al., 2018).

14-3-3 protein theta has also been introduced as hub that previous studies have reported that over-expression of 14-3-3ζ is an early event in oral tumorigenesis and may have a significant role in its development and progression. Thus, 14-3-3ζ might serve as a potential therapeutic target for oral cancer (Matta et al., 2007).

Laminin γ^2^ (LAMC2) is a subunit of the heterotrimeric glycoprotein laminin-332 that is a major component of epithelial basement membranes and play an important role in regulation of cell motility and adhesion (Marinkovich, 2007). It has been reported that LAMC2 was overexpressed in various carcinomas (Koshikawa et al., 1999; Yamamoto et al., 2001; Takahashi et al., 2002).

The last two hub genes with low expression in oral cancer were including *ACTR3* and* MYH11*. *ACTR3 *is a member of the ARP2/3 complex. Previously, investigations in the field of metastasis has been focused on the actin cytoskeleton and high expression of actin-related protein (ARP) during metastasis. Arp2/3 subunits have been reported by immunohistochemistry to be overexpressed in various cancer types, including lung (Semba et al., 2006), breast (Iwaya et al., 2007), gastric (Zheng et al., 2008) and colorectal cancers (Oser et al., 2009). Cortactin interacts with the Arp2/3 complex that there is a study showing that overexpression of cortactin in patients is related to high grade tumors, metastasis and poor survival in squamous cell carcinomas (Yamada et al., 2010; Sugahara et al., 2011).

The latest down-regulated hub gene was myosin-11 (*MYH11*), which is a smooth muscle myosin belonging to the myosin heavy chain family. Myosin MYH11 were previously found to be associated with variety of cancers, such as oral squamous cell carcinoma, meanwhile, a recent study has described *MYH11* as a potential biomarker and candidate drug target for head and neck cancer management (Islam et al., 2018). Above all, these results support our findings that these hub genes are involved in the pathogenesis of carcinoma by affecting cell proliferation, cell migration, and metastasis.

Increasing evidence has indicated that the dysregulation of miRNAs play a significant role in the pathogenesis of variety of cancer types, including OSCC. In this study, we screened top 10 DEMs, including 5 up-regulated and 5 down-regulated miRNAs in OSCC. Has-miR-5580-3p is one of the most significantly down-regulated miRNAs and was found to target hub genes including *CD44, ACLY, ACTR3, STAT1, YWHAZ* and* LAMC2*. Additionally, has-miR-1290 is the main up-regulated miRNA that potentially targets *ACTR3, MYH11*, and* YWHAZ*. A recent report shows that has-miR-5580-3p is down-regulated in OSCC samples, esophagus squamous cell carcinoma tissues and colorectal cancer compared with healthy control (Wang et al., 2017; Chamorro-Petronacci et al., 2018b; Cheng et al., 2019). Has-miR-1290, which is one of the most frequently and consistently up-regulated miRNAs in human cancer, has been suggested as crucial drivers for tumor initiation and cancer progression (Zhang et al., 2016), and play an oncogenic role in cellular processes of esophageal squamous cell carcinoma (ESCC) (Li et al., 2015). Recently, Zhang et al., reported that direct inhibition of Has-miR-1290 with locked nucleic acid administered systemically, can arrest the growth of established patient-derived xenograft tumours, implying that this miRNA is clinically helpful as potential biomarker for follow up disease progression and as therapeutic target (Zhang et al., 2016). Furthermore, research observations indicated that, miR-1290 can behave as non-invasive biomarkers that could be used as early detection, prognostic and diagnostic biomarker for broad spectrum of cancers (Mo et al., 2015; Kobayashi et al., 2018). In case of oral cancer, Nakashima et al. found that miR-1290 expression level was significantly lower in the plasma of oral squamous cell carcinoma patients than in that of healthy, however, circulating miR-1290 status suggested as useful biomarker to predict the clinical response to chemoradiotherapy as well as overall survival in oral squamous cell carcinoma patients (Nakashima et al., 2019). As we found that most of hub genes (including *CD44, ACLY, ACTR3, STAT1, LAMC2* and *YWHAZ*) were potentially targeted by has-miR-5580-3p, it indicates that this miRNA might play a key role in OSCC. In addition, according to Table 3, *STAT1* was targeted by three miRNAs including has-miR-6825-5P, has-miR-4495 and has-miR-5580-3P, indicating that these miRNAs play a significant role in OSCC development by mediating *STAT1*.

In conclusion, our study attempted to identify DEGs using comprehensive bioinformatics analyses and reported potential biomarkers for predicting the diseases progression. Our analysis has provided new points into OSCC pathogenesis by analyzing the hub genes and their interactions with miRNAs and consequently presenting the gene expression pattern, which can serve as candidate biomarkers and targets for potential treatment of OSCC. The limitation of this work is the lacking of verification experiments that could validate these predicted results obtained from bioinformatics analysis by using procedures such as qRT-PCR and Western Blot.
